# Seipin Deficiency Accelerates Heart Failure Due to Calcium Handling Abnormalities and Endoplasmic Reticulum Stress in Mice

**DOI:** 10.3389/fcvm.2021.644128

**Published:** 2021-03-11

**Authors:** Xiaoyue Wu, Xuejing Liu, Huan Wang, Zihao Zhou, Chengzhi Yang, Zijian Li, Youyi Zhang, XiaoLu Shi, Ling Zhang, Yuhui Wang, Xunde Xian, George Liu, Wei Huang

**Affiliations:** ^1^Institute of Cardiovascular Sciences and Key Laboratory of Molecular Cardiovascular Sciences, Ministry of Education, School of Basic Medical Sciences, Peking University Health Science Center, Beijing, China; ^2^Institute of Vascular Medicine, Peking University Third Hospital, Beijing, China; ^3^Experimental Research Center, China Academy of Chinese Medical Science, Beijing, China

**Keywords:** seipin, diastolic heart failure, calcium handling, endoplasmic reticulum stress, SERCA2a

## Abstract

*Seipin* deficiency can induce hypertrophic cardiomyopathy and heart failure, which often leads to death in humans. To explore the effects and the possible mechanisms of *Seipin* deficiency in myocardial remodeling, *Seipin* knockout (SKO) mice underwent transverse aortic constriction (TAC) for 12 weeks. We found a more severe left ventricular hypertrophy and diastolic heart failure and increases in inflammatory cell infiltration, collagen deposition, and apoptotic bodies in the SKO group compared to those in the wild type (WT) group after TAC. Electron microscopy also showed a more extensive sarcoplasmic reticulum expansion, deformation of microtubules, and formation of mitochondrial lesions in the cardiomyocytes of SKO mice than in those of WT mice after TAC. Compared with the WT group, the SKO group showed increases in endoplasmic reticulum (ER) stress-, inflammation-, and fibrosis-related gene expression, while calcium ion-related factors, such as *Serca2a* and *Ryr*, were decreased in the SKO group after TAC. Increased levels of the ER stress-related protein GRP78 and decreased SERCA2a and P-RYR protein levels were detected in the SKO group compared with the WT group after TAC. Slowing of transient Ca^2+^ current decay and an increased SR Ca^2+^ content in myocytes were detected in the cardiomyocytes of SKO mice. Adipose tissue transplantation could not rescue the cardiac hypertrophy after TAC in SKO mice. In conclusion, we found that *Seipin* deficiency could promote cardiac hypertrophy and diastolic heart failure after TAC in mice. These changes may be related to the impairment of myocardial calcium handling, ER stress, inflammation, and apoptosis.

## Introduction

Congenital generalized lipodystrophy (CGL) is an autosomal recessive disorder characterized by the severe loss of adipose tissue (AT), severe insulin resistance (IR), hypertriglyceridemia, fatty liver, renal injury, and cardiac hypertrophy (1). *Seipin* gene deficiency results in CGL type 2, which seems to cause the most severe lipodystrophy phenotype among CGLs in humans (2).

Several clinical studies have shown that hypertrophic cardiomyopathy is an important cause of heart failure and neonatal death in patients with CGL (3). A total of 20–25% of patients with CGL had cardiac hypertrophy, and the average age of onset was 20 years old (4). Another study found that *Seipin* gene deficiency underlying CGL2 was more likely to cause heart failure and premature death in premature infants than CGL1, and 42.9% of CGL2 patients had cardiomyopathy (5, 6). However, the exact mechanism of *Seipin* in cardiac remodeling is not clear.

SEIPIN is an intrinsic endoplasmic reticulum protein with two transmembrane structures (7). It has been reported that the SEIPIN-EGFP fusion protein colocalizes with the endoplasmic reticulum-specific protein calreticulin (4, 8, 9). Mutation or deletion of *Seipin* could result in endoplasmic reticulum stress (10, 11). Endoplasmic reticulum (ER) stress is involved in the occurrence and development of heart failure (HF) (12). Excessive or prolonged ER stress could lead to ion exchange disorders of the ER, inflammatory infiltration, and cell apoptosis (13). In this study, we investigated whether *Seipin* deficiency caused ER stress that is implicated in hypertrophic cardiomyopathy and HF. *Seipin* may regulate the distribution of intracellular calcium through the sarco/endoplasmic reticulum Ca^2+^-ATPase (SERCA) pump in adipocytes (14). Ca^2+^ plays an important role in myocardial contraction. SERCA2a is responsible for the translocation of Ca^2+^ from the cytoplasm to the ER during myocardial diastole. The patients with diastolic HF had abnormal SERCA2a function (15). Therefore, hypertrophic cardiomyopathy and HF caused by *Seipin* deficiency may be associated with a decrease in SERCA2a function. However, no evidence of cardiac hypertrophy was detected in *Seipin* knockout (SKO) mice generated by us until the age of 9 months. The mice presented only mild cardiac hypertrophy at 9–12 months of age. Therefore, we used a pressure overload model with transverse aortic constriction (TAC) to study the effect of *Seipin* on cardiac hypertrophy in SKO mice.

In this study, we found that *Seipin* gene deficiency can accelerate cardiac remodeling in animal models, leading to cardiac hypertrophy and diastolic heart failure. Further studies indicated that these changes may be related to the impairment of myocardial calcium handling, endoplasmic reticulum stress, inflammation, and apoptosis.

## Materials and Methods

### Animals

The animal experiments were approved by the Institutional Animal Care Research Advisory Committee of the National Institute of Biological Science (NIBS) and the Animal Care Committee of Peking University Health Science Center, and the study was approved by the local Ethical Committee. Animals were maintained on a normal diet (10% of kilocalories were obtained from fat) and a 12-h light/12-h dark cycle with free access to water. The Principles of Laboratory Animal Care (NIH Publication, 8th Edition, 2011) were followed.

SKO and littermate wild type (WT) male mice with a C57BL/6 background at the age of 2 months were used in this study. SKO mice were generated and bred as described previously (16). Animals were divided into four groups (*n* = 8 for each group): The WT and SKO sham operation groups and the WT-and SKO-TAC operation groups. Echocardiography was performed every 2 weeks. Mice were sacrificed at 12 weeks after TAC. The heart weight to tibia length (HW/TL) ratios were determined. Samples were obtained from the heart, and some were immediately frozen in liquid nitrogen and stored at −80°C for real-time PCR, whereas some were fixed for histological studies.

To study whether AT is involved in cardiac hypertrophy in SKO mice, AT transplantation was performed 2 weeks before TAC surgery at the age of 6 weeks. The animals were divided into four groups (*n* = 8): SKO-TAC and WT-TAC mice transplanted with the AT of WT mice; SKO-TAC and WT-TAC mice underwent sham surgery. The animals were sacrificed 12 weeks after TAC, and the aforementioned parameters were analyzed.

### Transverse Aortic Constriction

To investigate the impact of pressure overload-induced cardiac hypertrophy, transverse aortic constriction (TAC) surgery was performed in mice as described previously (17, 18). Briefly, mice were anesthetized with 2% isoflurane (Baxter Healthcare Corporation, New Providence, NJ, USA) and then maintained on 1.5% isoflurane. Mice were ventilated by tracheal intubation using a rodent ventilator (Alcbio Corporation, Shanghai, China) with a respiratory rate of 120 breaths/min and a tidal volume of 0.3 mL. Then, the chest was opened at the suprasternal fossa along the midsternal line, and the thymus glands were superiorly reflected. The transverse thoracic aorta between the innominate artery and the left common carotid artery was dissected, and a 6-0 silk suture was tied around the aorta, which was pressed against a 26-gauge needle. Then, the needle was removed. A sham operation involving thoracotomy and aortic dissection without constriction of the aorta was performed in both the WT and SKO sham operation groups.

### Echocardiography

Cardiac hypertrophy and function were assessed by echocardiography using a high-resolution Vevo 770TM Imaging System (Visual Sonics Inc., Toronto, Canada) as described previously (19, 20).

Mice were anesthetized with 3% isoflurane (Baxter Healthcare Corp, New Providence, RI, USA), and their chests were shaved. The bodies of the mice were placed in the supine position on a movable heated platform that was maintained at 37°C. Then, the mice were anesthetized with 1.0–1.5% isoflurane to keep the heart rate stabilized at 400–500 beats per min. At the level of the papillary muscle of the heart in mice, two-dimensional parasternal long-axis views and short-axis views were obtained. The M-mode cursor was positioned perpendicular to the maximum LV dimension during end-diastole and systole. The following variables were measured digitally in the M-mode images: LV (left ventricular) internal dimensions (LVID); LV anterior wall thickness (LVAW); LV posterior wall thickness (LVPW). The values of the LV end-diastolic volume (LVEDV), ejection fraction (EF) were calculated from these parameters.

Pulsed Doppler ultrasound was used to measure the velocity of the transmitral flow of the ventricular filling. The early diastolic mitral transmitral flow velocity (E), late diastolic mitral transmitral flow velocity (A), and the E/A ratio were used to assess diastolic function. The normal value was >1, and an inverse E/A ratio was detected in diastolic HF. The early diastolic velocity (Em) and the late diastolic velocity (Am) of the mitral valve ring and Em/Am were also determined based on the ultrasound scan. All measurements represented the average value from three continuous cardiac cycles per loop.

### RNA Isolation and Quantitative Real-Time PCR

Total RNA from tissues (~50–100 mg of each tissue) was extracted using Tri Reagent (Molecular Research Center, OH, USA), and first-strand cDNA was generated by using an RT kit (ABM, Richmond, BC, Canada). Quantitative real-time PCR was performed using the primers listed in Table 1. Amplifications were performed with 35 cycles using a continuous fluorescence detection system (Agilent Technologies, Santa Clara, Cal, USA) with EVA Green fluorescence reagent (ABM, Richmond, BC, Canada). Each cycle consisted of denaturation by heating for 15 s at 95°C and annealing/extension for 60 s at 60°C. All samples were quantified using the comparative CT method for relative quantification and normalized to GAPDH (21). Each sample was measured in triplicate. Melting curve analysis was used to confirm the amplification specificity. All experiments were repeated at least twice in triplicate.

**Table 1:** Primers used in real-time polymerase chain reaction.

**Genes**	**Sense primers**	**Antisense primers**
*Anp*	GGGCTTCTTCCTCGTCTTGG	GGGGGCATGACCTCATCTTC
*Bnp*	TAACGCACTGAAGTTGTTGTAGG	CGCTATGTTTATTATGTTGTGGC
*β-mhc*	CTTCACTCCAGAAGAGAAGAACTC	CCCATGAGGTAGGCTGATTTG
*Myh7*	ACTGTCAACACTAAGAGGGTCA	GGATGATTTGATCTTCCAGGG
*Myh6*	GCCCAGTACCTCCGAAAGTC	GCCTTAACATACTCCTCCTTGTC
*Il6*	TTCTTGGGACTGATGCTG	CTGGCTTTGTCTTTCTTGTT
*Icam1*	GGACCTTAACAGTCTACAAC	AGCCGAGGACCATACA
*Serca2*	GAGAACGCTCACACAAAGACC	CAATTCGTTGGAGCCCCAT
*Col3a1*	CCTGGCTCAAATGGCTCAC	CAGGACTGCCGTTATTCCCG
*Col1a2*	GTAACTTCGTGCCTAGCAACA	CCTTTGTCAGAATACTGAGCAGC
*Xbp1*	AAGAACACGCTTGGGAATG	AGGGAGGCTGGTAAGGAA
*Ryr*	GCCTTATCCATGCCCAGTAA	CCAGTGGGACAGACTGGTGA
*Ncx1*	TGGTAGATGGCAGCAATGGA	CTTCGTCCCACCTACAGAAT
*Seipin*	TCAATGACCCACCAGTC	AAGGAGCCATAGAGGAAC
*Bax*	CCAGGATGCGTCCACCAA	AAGTAGAAGAGGGCAACCAC
*Bcl2*	TACCGTCGTGACTTCGCAGAG	GGCAGGCTGAGCAGGGTCTT
*Grp78*	ACTTGGGGACCACCTATTCCT	GTTGCCCTGATCGTTGGCTA
*Gapdh*	TGATGACATCAAGAAGGTGGTGAAG	TCCTTGGAGGCCATGTAGGCCAT

*Anp, Atrial natriuretic peptide; Bnp, B-type natriuretic peptide; β-mhc, Beta-Myosin Heavy Chain; Myh7, Myosin heavy chain7; Myh6, Myosin heavy chain6; Il6, Interleukin-6; Icam1, Intercellular adhesion molecule-1; Serca2, Sarco/endoplasmic reticulum Ca^2+^-ATPase2; Col3a1, Collagen type III alpha 1 chain; Col1a2, Collagen type I alpha 2; Xbp1, X-box binding protein 1; Ryr, Ryanodine receptor; Ncx1, Na^+^/Ca^2+^ exchanger 1; Bax, BCL-2 Associated X; Bcl2, B-cell lymphoma 2; Grp78, 78-kDa glucose-regulated protein; Gapdh, Glyceraldehyde-3-phosphate dehydrogenase*.

### Western Blotting

Myocardial tissues were homogenized in RIPA buffer, and the protein content was determined by a BCA protein assay (Thermo Fisher Scientific, USA) as previously described (16, 21). The antibodies that were used (Abcam, Cambridge, MA, USA) were diluted at 1:1,000 and were as follows: SERCA2a antibody (ab3625), glucose regulated protein 78 (GRP78) antibody (ab21685), ryanodine receptor-2 (RYR2) antibody (ab2868), ryanodine receptor-phospho (RYR-P) antibody (ab59225), and Na^+^/Ca^2+^ exchanger (NCX) antibody (ab2869). The protein bands were analyzed using densitometry by an imaging system (molecular imager, ChemiDoc XRS, Bio-Rad, USA). The examined protein levels were normalized to that of GAPDH. The results are represented by the ratios of the values of the experimental groups to those of the WT control group for GRP78, RYR, and SERCA2a.

### Cardiac Histological and Morphometric Analysis

Animals were anesthetized with 1% pentobarbital (45 mg/kg) and cardinally perfused with cold PBS. The hearts were cut transversally, fixed in 4% neutral formalin, and embedded in paraffin. The 3 μm-thick sections were cut from the paraffin-embedded hearts. Sections were stained with hematoxylin-eosin (HE) and Sirius Red to analyze morphology and fibrosis (16, 22). To assess the cardiomyocyte cross-sectional area, the sections were stained with wheat germ agglutinin (WGA) as previously described (23). The body weight, heart weight, and tibia length were also measured.

### Electron Microscopy

The specimens from the cardiac ventricles were fixed in 2.5% glutaraldehyde, post-fixed in 1% osmium tetroxide, dehydrated in serial acetone solutions, and embedded in Epon 812 resin. The ultrathin sections were stained with uranyl acetate and lead citrate solutions and then visualized with a JEOL 1230 transmission electron microscope (JEOL, Tokyo, Japan).

### Isolation of Ventricular Myocytes

Mouse LV myocytes were isolated enzymatically by a protocol described previously (24). One hour after the myocyte pellet was suspended in KB solution, the cells were initially washed with Ca^2+^-free Tyrode's buffer to remove the KB solution, and extracellular Ca^2+^ was slowly added to bring the concentration back up to 1.8 mM. Only calcium-tolerant, quiescent, and rod-shaped cells showing clear cross striations were used for Ca^2+^ and sarcomere shortening measurements.

### Transient Cytosolic Ca^2+^ and Sarcomere Shortening Measurements

Myocytes were loaded with 2 μM fura-2AM for 20 min, and fluorescence measurements were recorded with a dual-excitation fluorescence photomultiplier system (IonOptix, North America, Milton, Massachusetts, USA). After loading, the cells were washed and resuspended in Tyrode's solution and placed in the cell chamber, in which they were stimulated for 1–4 ms; then, they were superfused at 37°C and imaged with a Fluor-40 objective. Myocytes were then exposed to light that was emitted by a 75 W xenon lamp and passed through either a 340- or 380-nm wavelength filter. The emitted fluorescence was detected at 510 nm. The background fluorescence of each myocyte was determined by moving the myocyte out of the view and recording the fluorescence of the solution alone. Cell shortening was synchronously measured *via* transient calcium currents in myocytes. Soft Edge software (IonOptix, North America, Milton, Massachusetts, USA) was used to capture and analyze the changes in sarcomere length during cell shortening and lengthening.

### Caffeine-Sensitive Ca^2+^ Release

Caffeine-sensitive Ca^2+^ release was assessed as described by Bassani et al. (25) to assess the SR (sarcoplasmic reticulum) calcium content and the contributions of SERCA and NCX to diastolic calcium removal. Myocytes were perfused with Tyrode's solution and stimulated at 1 Hz. Rapid application of 20 mM caffeine was employed to induce SR Ca^2+^ release and assess the contributions of NCX and the slow transport systems (mitochondrial Ca^2+^ uniporter and sarcolemmal Ca^2+^-ATPase). Rapid changes in the superfusate were achieved using an ALA quick switch system to rapidly change the solution bathing the cell, thus allowing us to assess the immediate effect of caffeine on cell contracture. The decrease in fluorescence (F340/380) during caffeine-induced Ca^2+^ transient currents was attributable to NCX and slow transport system mechanisms. Because slow mechanisms remove only 0.5% of Ca^2+^ in mice, the contributions of these mechanisms are negligible (26). The decline in fluorescence during stimulated twitches was attributable to both SERCA and NCX. The contribution of SERCA was therefore evaluated as the difference between the decline in fluorescence during caffeine-induced Ca^2+^ transient currents and the decline in fluorescence during electrically stimulated twitches.

### AT Transplantation and Plasma Biochemical Determination

Mouse AT was transplanted as described previously (16, 21). Animals were anesthetized with 1% pentobarbital sodium (45 mg/kg). Donor subcutaneous AT was divided into 100–150-mg pieces. A total of six to eight pieces of 900-mg grafts were implanted subcutaneously in the SKO or WT mice through incisions in the shaved skin of the back. Incisions were closed using 5-0 silk sutures. The SKO and WT control mice received the same operation except for AT transplantation. After surgery, the mice were housed individually for 1 week and then at 3–4 mice/cage.

Plasma insulin, leptin, and adiponectin levels were measured by ELISA (Linco Research). For glucose tolerance tests (GTT), mice that fasted for 4 h were given i.p. glucose (2 g/kg body weight; Abbott), and blood samples were collected before (time 0) and at 15, 30, 60, and 120 min after injection for glucose measurement (16, 21).

### Statistical Analysis

All data are presented as the means ± SEM. Statistical comparisons between groups were performed using the 2-way ANOVA followed by the Tukey test or the Mann-Whitney *U*-test for non-parametric analysis. A value of *P* < 0.05 was considered statistically significant.

## Results

### The Seipin Gene Was Expressed in the Heart

**Figure 1:**
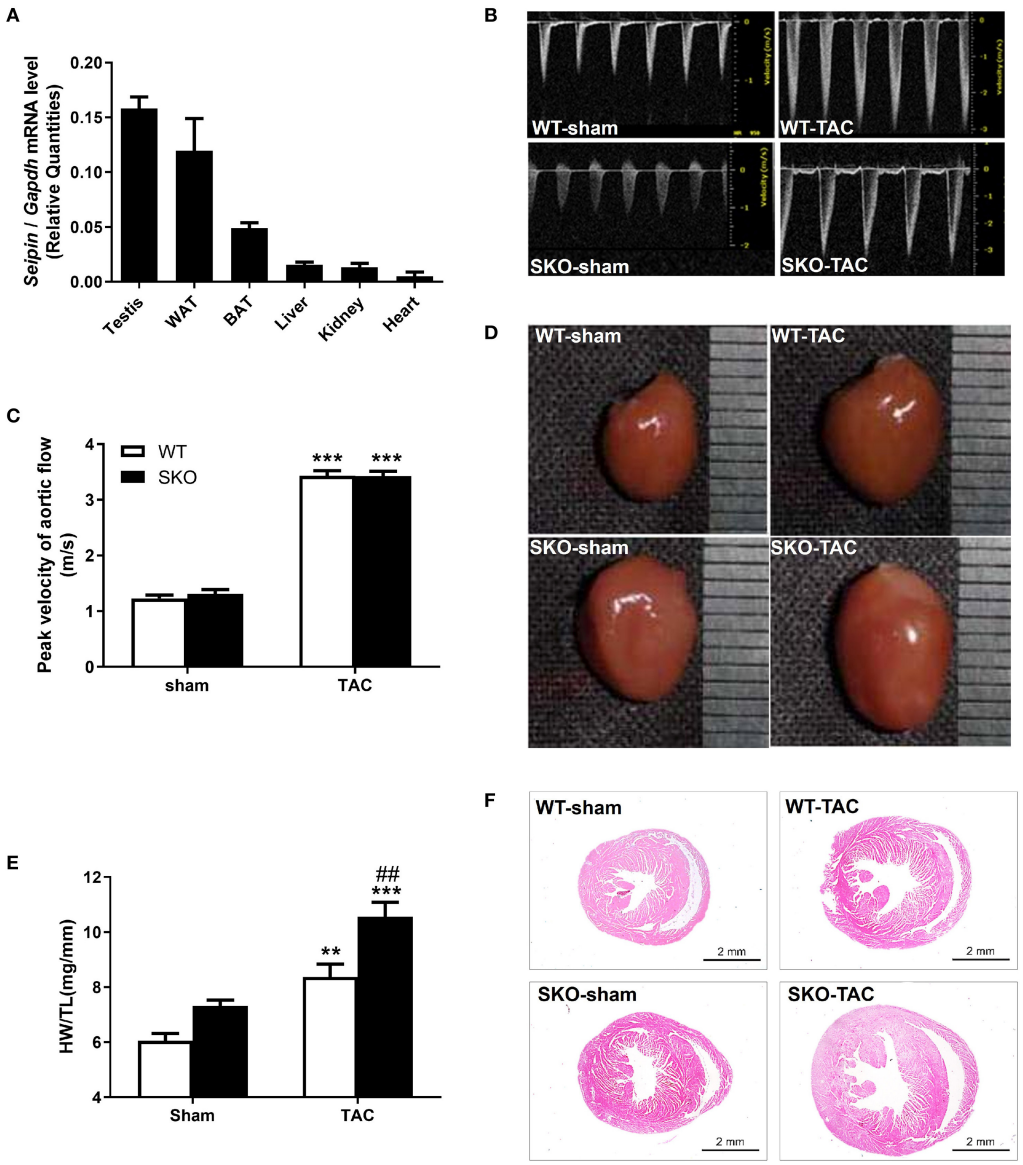
*Seipin* mRNA expression in WT mice and *Seipin* gene deficiency promoted myocardial hypertrophy at 12 weeks after TAC. **(A)** *Seipin* mRNA expression levels in different tissues in 6-month-old C57BL/6 WT male mice; **(B)** Blood flow spectrum of the aorta; **(C)** Peak values of the blood flow velocity; **(D)** Representative photographs of the heart; **(E)** Ratio of heart weight/tibia length (HW/TL); **(F)** Hematoxylin–eosin staining of longitudinal sections of the heart. **P < 0.01, ***P < 0.001, effect of TAC; ^##^*P* < 0.01, effect of genotype. WAT, white adipose tissue; BAT, brown adipose tissue. WT, Wild-type; SKO, *Seipin* knockout; TAC, transverse aortic constriction.

Six-month-old C57BL/6 WT mice were used to analyze *Seipin* mRNA expression in different tissues (*n* = 8). *Seipin* was abundantly expressed in testis and AT, as described previously (16, 21). We also found a low mRNA expression level of *Seipin* in the heart, which was 10% of that in the testis (Figure 1A).

### Seipin Gene Deficiency Promoted Myocardial Hypertrophy After TAC

SKO mice showed only mild cardiac hypertrophy until the age of 9 months (HW/BW: WT: 5.18 ± 0.41; SKO: 5.97 ± 0.78; *P* < 0.05). Therefore, we established a pressure overload myocardial remodeling model to accelerate cardiac remodeling by performing TAC surgery. The velocity of blood flow in the aortic arch ligation site was detected 3 days after the surgery and was found to be increased about 3-fold compared with that in the sham group (Figures 1B,C; *P* < 0.001). There was no significant difference concerning the flow velocity between SKO and WT mice before or after surgery (Figures 1B,C). These results indicate that the TAC model was successfully established, and there was no effect of genotypes on the flow velocity at the ligation site.

The result of the whole heart images showed the enlargement of the heart in WT and SKO mice at 12 weeks after TAC, and SKO mice had a more severe phenotype (Figure 1D). Significantly increased HW/TL ratios were detected in WT and SKO mice after TAC than in the sham groups (Figure 1E; *P* < 0.01 and *P* < 0.001, respectively). The HW/TL in SKO mice were significantly higher than those in WT mice after TAC (Figure 1E; *P* < 0.01). The heart tissue cross-sections showed alterations similar to those noted in Figures 1D–F.

### Seipin Deficiency Induced Severe Cardiac Hypertrophy and LV Dysfunction After TAC

**Figure 2:**
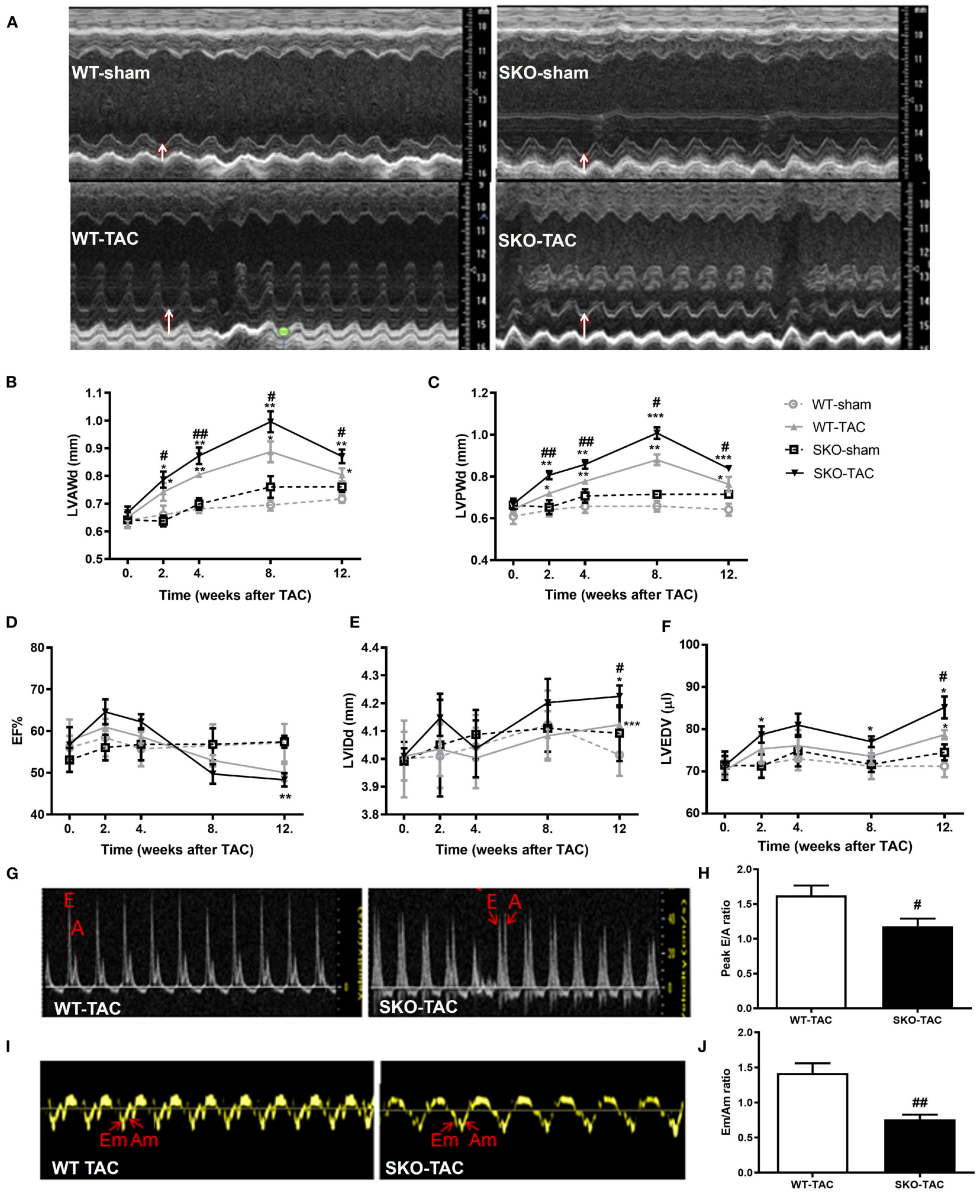
*Seipin* deficiency induced severe cardiac hypertrophy and left ventricular dysfunction at 12 weeks after TAC by echocardiography. **(A)** Representative M-mode echocardiographs; white arrows represent the left ventricular posterior wall thickness (LVPWd); **(B)** left ventricular anterior wall thickness in diastole (LVAWd); **(C)** LVPWd; **(D)** ejection fraction (EF%); **(E)** left ventricular internal diameter in diastole (LVIDd); **(F)** left ventricular end-diastolic volume (LVEDV); **(G)** Representative transmitral Doppler flow profile; **(H)** Peak E/A ratio; **(I)** Representative Doppler images of the basal inferolateral LV wall; and **(J)** Em/Am ratio.*P < 0.05, **P < 0.01, ***P < 0.001, effect of TAC; ^#^*P* < 0.05, ^##^*P* < 0.01, effect of genotype. WT, Wild-type; SKO, *seipin* knockout; TAC, transverse aortic constriction, late diastolic mitral transmitral flow velocity (A), early diastolic mitral transmitral flow velocity (E), late diastolic velocity (Am), and early diastolic velocity (Em).

Echocardiography was performed at 2, 4, 8, and 12 weeks after TAC. The LVAW in diastole (d) and LVPWd were increased in both WT and SKO mice 2 weeks after TAC and reached a peak at 8 weeks. The LVAWd and LVPWd were greater in SKO-TAC mice than in WT-TAC mice at 2 weeks (Figures 2A–C; *P* < 0.05 and *P* < 0.01, respectively); they were increased by 12.2–14.5% at 8 weeks and by 8.3–6.23% at 12 weeks in SKO-TAC mice compared with those in WT-TAC mice, respectively (Figures 2B,C). The EF% was decreased at 12 weeks in SKO-TAC mice compared with that in SKO-sham mice (Figure 2D; *P* < 0.01). In contrast, no significant difference in the EF% was observed between SKO-TAC and WT-TAC mice (Figure 2D). The LVIDd and LVEDV were increased in both the SKO-TAC and WT-TAC groups compared with those in the sham groups at 12 weeks (Figures 2E,F), and the LVIDd in the SKO-TAC group was 7.3% higher than that in the WT-TAC group (Figure 2E; *P* < 0.05). Meanwhile, the LVEDV in the SKO-TAC group was 8.5% higher than that in the WT-TAC group at 12 weeks (Figure 2F; *P* < 0.05). The results of the evaluation of cardiac diastolic function showed that the E/A and Em/Am peaks were inversed (Figures 2G–J); that is, the ratios of E/A and Em/Am were <1.0 in SKO-TAC mice, and were decreased significantly than in WT-TAC mice (Figures 2H–J; *P* < 0.05 and *P* < 0.01, respectively). This indicated that *Seipin* deficiency promoted diastolic HF after TAC in mice. Compared with WT mice, SKO mice showed a more obvious left ventricular hypertrophy and diastolic HF at 12 weeks after TAC.

### Seipin Deficiency Accelerated Cardiomyocyte Hypertrophy, Inflammation, and Fibrosis in the Heart After TAC

**Figure 3:**
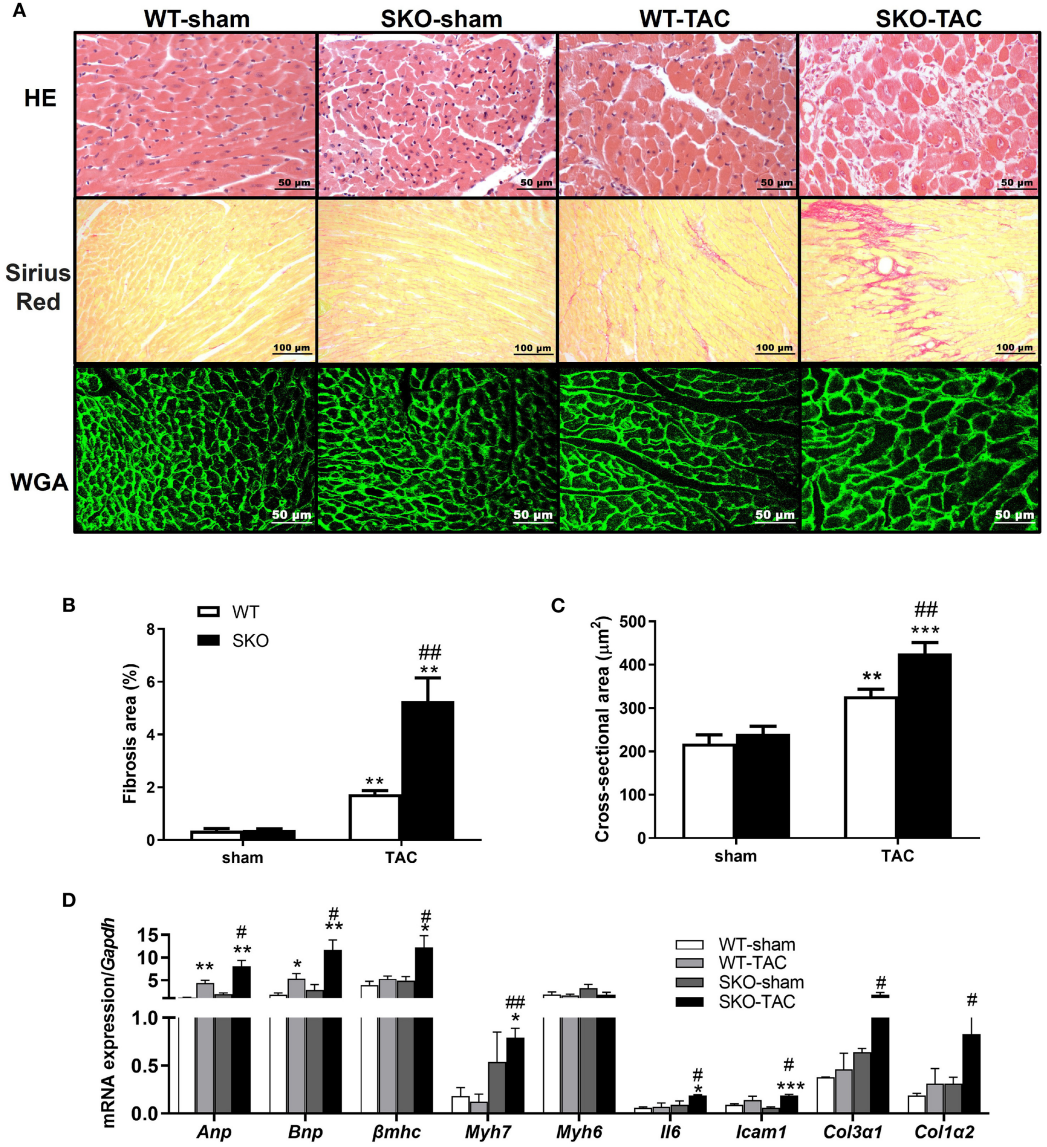
*Seipin* deficiency accelerated cardiomyocyte hypertrophy and inflammation and fibrosis in the heart after TAC. **(A)** Hematoxylin-eosin (HE), Sirius red and wheat germ agglutinin (WGA) staining of the heart cross-section; **(B)** Percentage of fibrosis area in heart tissue by Sirius red staining; **(C)** Quantification of cardiomyocyte cross-sectional area by WGA staining; **(D)** mRNA expression levels of *Anp, Bnp*, β*mhc, Myh7, Myh6, Il6, Icam1, Col3*α*1*, and *Col1*α*2*. *P < 0.05, **P < 0.01, ***P < 0.001, effect of TAC; ^#^*P* < 0.05, ^##^*P* < 0.01, effect of genotype. WT, Wild-type; SKO, *Seipin* knockout; TAC, transverse aortic constriction.

Compared with the WT-TAC group, the SKO-TAC group showed a more extensive myocardial fiber disarray and fracture and increased inflammatory cell infiltration in the heart by HE straining (Figure 3A). Sirius red staining showed increased fibrosis areas in both WT (Figures 3A,B; *P* < 0.01) and SKO (Figures 3A,B; *P* < 0.01) mice after TAC, moreover, fibrosis areas were increased in SKO-TAC mice compared with WT-TAC mice (Figures 3A,B; *P* < 0.01). The cross-sectional area of myocytes was measured in WGA stained LV myocardium. The mean cardiomyocyte cross-sectional areas were increased in both WT and SKO mice after TAC (Figures 3A–C; *P* < 0.01 and *P* < 0.001, respectively). Moreover, the cardiomyocyte cross-sectional areas were significantly greater in the SKO-TAC group than those in the WT-TAC group (Figures 3A–C; *P* < 0.01).

The relative expression levels of heart failure related genes, such as *Anp* and *Bnp*, were significantly increased after TAC in both the WT (Figure 3D; *P* < 0.01 and *P* < 0.05, respectively) and SKO (Figure 3D; *P* < 0.001 and *P* < 0.01, respectively) groups and were higher in the SKO-TAC group than in the WT-TAC group (Figure 3D; *P* < 0.05). The relative expression levels of cardiac hypertrophy-related genes, such as β*mhc* and *Myh7*, were increased in both groups after TAC and were higher in the SKO-TAC group than in the WT-TAC group (Figure 3D; *P* < 0.05 and *P* < 0.01, respectively). The relative expression levels of inflammatory factor genes, such as *Il6* and *Icam1*, were significantly higher in the SKO group after TAC (Figure 3D; *P* < 0.05 and *P* < 0.001, respectively) and were higher in the SKO-TAC group than in the WT-TAC group (Figure 3D; *P* < 0.05). The relative expression levels of fibrosis-related genes, such as *Col3*α*1* and *Col1*α*2*, were significantly increased in SKO-TAC mice compared with those in WT-TAC mice (Figure 3D; *P* < 0.05). Taken together, these results indicate that *Seipin* deficiency accelerated cardiomyocyte hypertrophy, inflammation, and fibrosis after TAC in mice.

### Seipin Deficiency Aggravated Myocardial Ultrastructure Changes by Increasing Endoplasmic Reticulum Stress and Apoptosis After TAC

**Figure 4:**
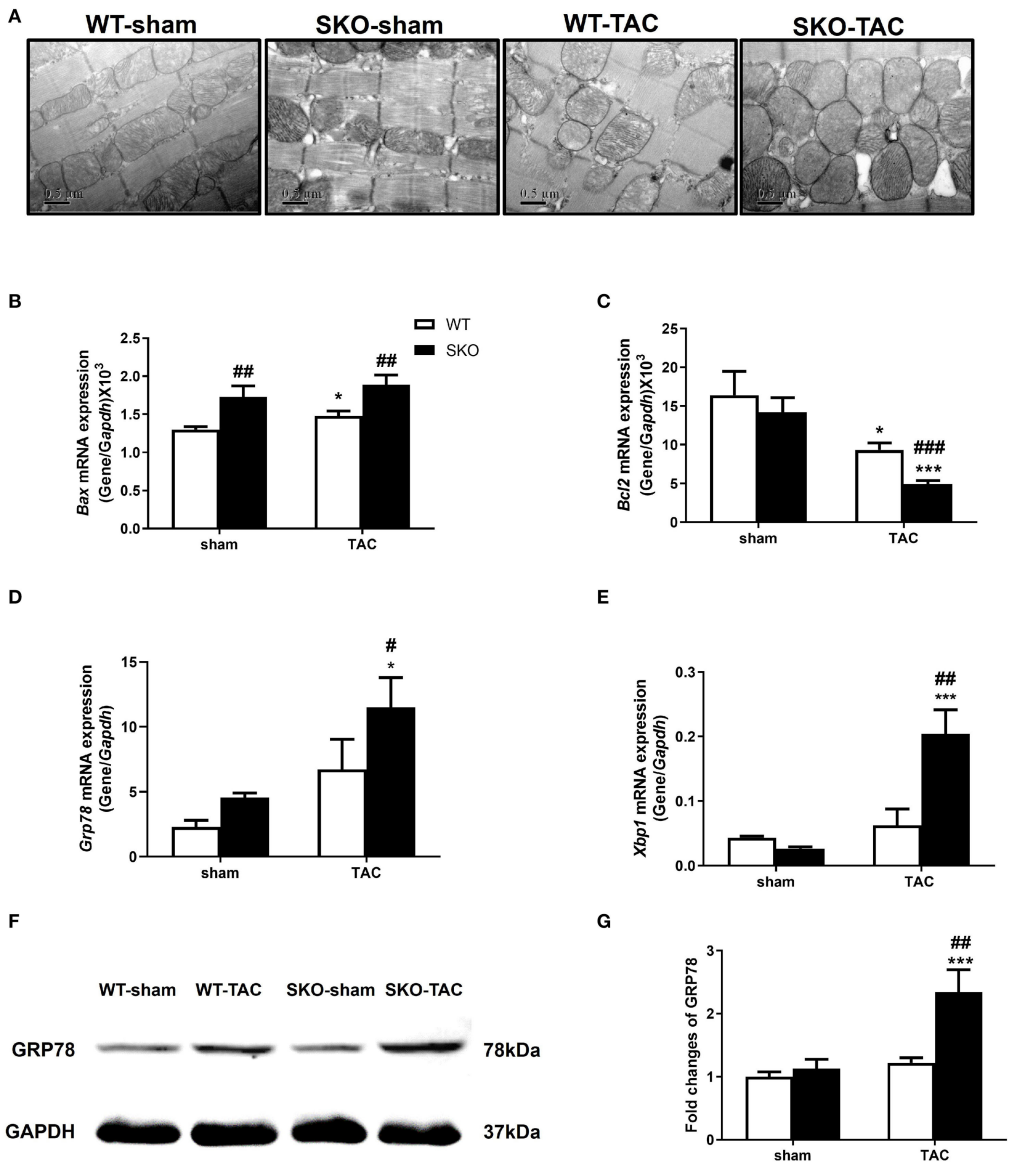
*Seipin* deficiency aggravated myocardial ultrastructure changes and increased endoplasmic reticulum stress and apoptosis after TAC. **(A)** Representative cardiac electron microscopy images (original magnification, 40,000×); **(B–E)** mRNA expression levels of *Bax, Bcl2, Grp78, and Xbp1*, in the heart; **(F,G)** Western blot analysis of GRP78 and densitometric quantitation. The results are represented by the ratio of the values of the experimental groups to those of the WT control group. *P < 0.05, ***P < 0.001, effect of TAC; ^#^*P* < 0.05, ^##^*P* < 0.01, ^###^*P* < 0.001, effect of genotype. WT, Wild-type; SKO, *Seipin* knockout; TAC, transverse aortic constriction.

Electron microscopy analysis detected hypertrophy and disordered arrangement of mitochondria in WT mice at 12 weeks after TAC. In addition to the previously described changes, there was hyperplasia, partial disappearance of the ridge, obvious sarcoplasmic reticulum dilatation, and compression deformation of the transverse tube (TT), which reduced the size of the transverse tube-sarcoplasmic reticulum (TT–SR) dyadic junctions in SKO-TAC mice (Figure 4A).

The relative gene expression levels of the apoptotic factor *Bax* were increased in WT after TAC (Figure 4B; *P* < 0.05) and were higher in SKO mice than in WT mice with or without TAC (Figure 4B; *P* < 0.01). The gene expression levels of the anti-apoptotic factor *Bcl2* were decreased in both WT and SKO mice after TAC (Figure 4C; *P* < 0.05 and *P* < 0.001, respectively) and were much lower in the SKO-TAC group than in the WT-TAC group (Figure 4C; *P* < 0.001).

We then detected the changes in the relative expression of ER stress factors in the hearts of SKO mice caused by pathological changes of the sarcoplasmic reticulum. The gene expression levels of *Grp78* were significantly increased in SKO-TAC mice compared with those in SKO and WT-TAC mice (Figure 4D; *P* < 0.05). The gene expression levels of *Xbp1* were significantly increased in SKO-TAC mice compared with those in SKO and WT-TAC mice (Figure 4E; *P* < 0.001 and *P* < 0.01, respectively). The protein levels of GRP78 were also elevated in the SKO-TAC group compared with that in the sham operation control (Figures 4F,G; *P* < 0.001) and the WT-TAC group (Figures 4F,G; *P* < 0.01). These results showed that *Seipin* deficiency induced myocardial ER stress in mice after TAC.

Taken together, these results revealed that *Seipin* deficiency induced obvious pathological changes in the mitochondria and sarcoplasmic reticulum in cardiomyocytes after TAC, which might be associated with ER stress and apoptosis.

### Seipin Deficiency Resulted in Abnormalities in Calcium Handling in Ventricular Myocytes

**Figure 5:**
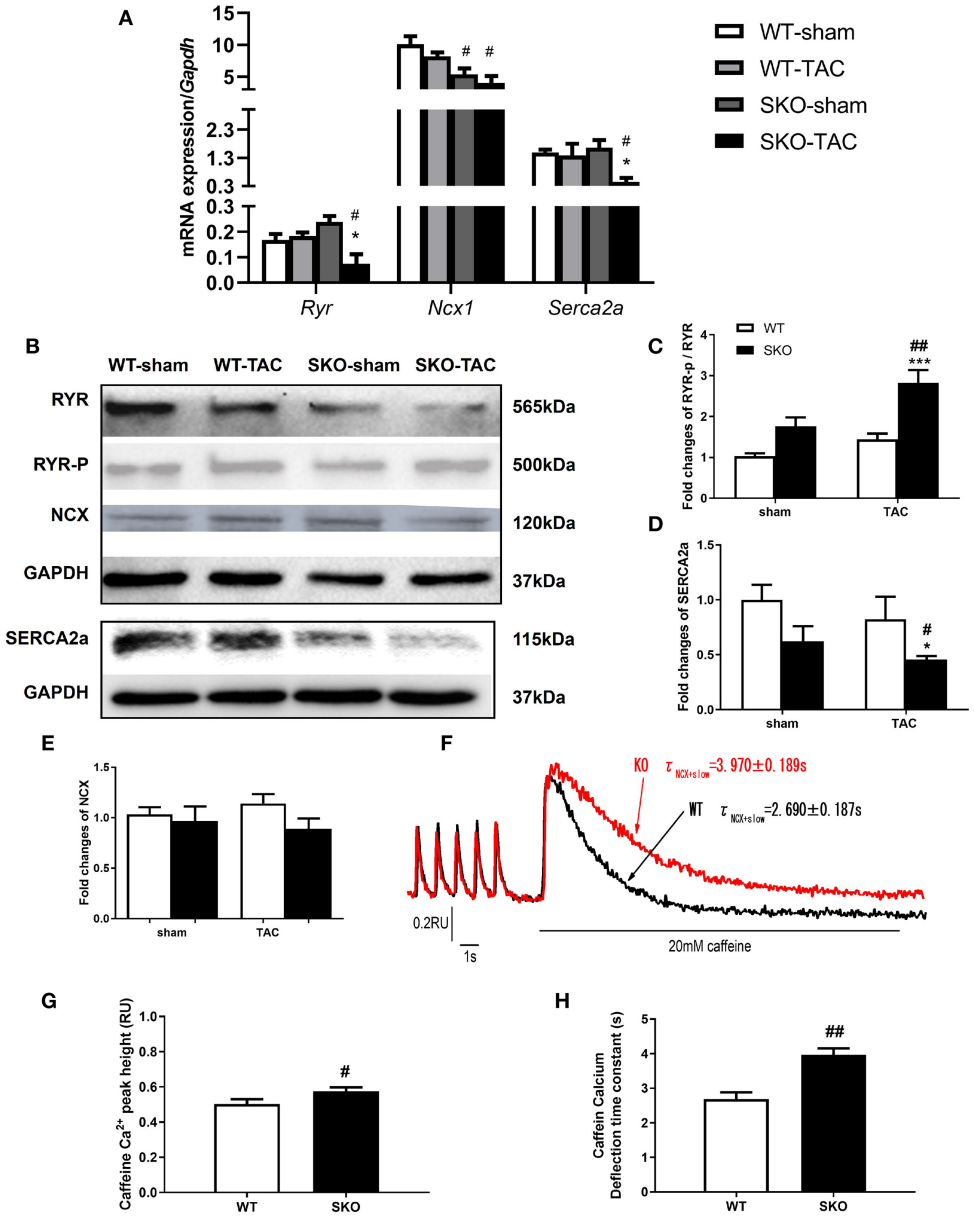
*Seipin* deficiency induced abnormalities in the calcium handling of ventricular myocytes. **(A)** mRNA expression levels of *Ryr, Ncx1*, and *Serca2a*; **(B–E)** Western blot analysis of RYR, SERCA2a, and NCX and densitometric quantitation; **(F)** Curve plot of stimulation-induced and caffeine-induced Ca^2+^ transient current fluorescence (F340/F380, RU); **(G)** Peak height of caffeine-induced Ca^2+^ release (indicated by the SR Ca^2+^ content); **(H)** Caffeine-induced Ca^2+^ transient current deflection time constant (taucaff) (indicating the functionality of NCX in removing diastolic Ca^2+^). The results are represented as the ratios of the values in the experimental groups to those of the WT control group for RYR and SERCA2a.*P < 0.05, ***P < 0.001, effect of TAC; ^#^*P* < 0.05, ^##^*P* < 0.01, effect of genotype. WT, Wild-type; SKO, *Seipin* knockout; TAC, transverse aortic constriction.

Ca^2+^ handling plays an important role in maintaining the function of myocytes (27). We therefore detected myocardial Ca^2+^ handling-related gene and protein expression. The gene expression levels of *Ryr* and *Serca2a* were significantly decreased in the SKO-TAC group compared with those in the SKO-sham and WT-TAC groups (Figure 5A; *P* < 0.05). The ratio of phospho-RYR to RYR was increased in SKO-TAC mice compared with those in SKO and WT-TAC mice (Figures 5B,C; *P* < 0.001 and *P* < 0.01, respectively). The protein levels of SERCA2a were considerably decreased in SKO mice after TAC (Figures 5B,D; *P* < 0.05) and were lower in SKO-TAC mice than in WT-TAC mice (Figures 5B,D; *P* < 0.05). There were no significant differences in NCX gene and protein expression levels among the four groups (Figures 5A,B,E).

To understand the alteration of Ca^2+^ handling and E-C coupling in ventricular myocytes, we measured Ca^2+^ transient currents and sarcomere shortening in SKO LV myocytes. We found that the Ca^2+^ transient current decline time constant (tau) was significantly lengthened in the SKO group compared with that in the WT group (Table 2). To understand the mechanism underlying the decrease in the capacity of Ca^2+^ decline in SKO myocytes, we assessed the caffeine-induced Ca^2+^ release peak height (phcaff) and diastolic Ca^2+^ removal by measuring the decline in fluorescence (F340/380) during caffeine-induced Ca^2+^ transient currents (taucaff) and Ca^2+^ transient currents during stimulated twitches (tautwitch) (at 1 Hz). Our results showed that taucaff was 2.690 ± 0.187 s for WT myocytes and 3.970 ± 0.189 s for SKO myocytes (Figure 5F; *P* < 0.01). In addition, phcaff was 0.50 ± 0.03 RU (ratio unit) for WT myocytes and 0.57 ± 0.02 RU for SKO myocytes (Figure 5G; *P* < 0.05). Slow Ca^2+^ transient current decay was also detected in SKO myocytes (Figure 5H; *P* < 0.01). The reduction in NCX function might have contributed to the slowing of Ca^2+^ transient current decay and the increase in SR Ca^2+^ content in SKO myocytes, which led to Ca^2+^ transport dysfunction in SKO ventricular myocytes (Figures 5F–H).

**Table 2:** Calcium transient currents and sarcomere shortening in ventricular myocytes of SKO mice; [Ca^2+^]i, cytosolic Ca^2+^ concentration.

	**WT (*n* = 39)**	**SKO (*n* = 64)**
**Calcium transients**		
Baseline, RU	0.81 ± 0.02	0.82 ± 0.01
Departure velocity	23.73 ± 1.16	24.76 ± 0.79
Peak height, RU	0.32 ± 0.01	0.31 ± 0.01
Time to peak, ms	33.60 ± 1.08	35.23 ± 1.09
Time to 50% peak, ms	−1.77 ± 0.09	−1.78 ± 0.06
Return velocity	10.51 ± 0.25	10.69 ± 0.27
${\text{Ca}}_{\text{i}}^{2+}$ decay time constant, ms	233.07 ± 7.79	267.15 ± 9.60^*^
**Sarcomere shortening**		
Baseline, μm	1.72 ± 0.01	1.75 ± 0.01
Departure velocity	−1.30 ± 0.07	−1.26 ± 0.07
Fractional shortening, %	3.09 ± 0.17	3.19 ± 0.17
Time to peak, ms	100.19 ± 4.17	102.71 ± 3.15
Time to 50% peak, ms	0.55 ± 0.05	0.60 ± 0.06
Return velocity	36.16 ± 1.54	38.09 ± 1.26
Time to 50% relaxation, ms	90.66 ± 4.91	89.29 ± 4.03

*WT, Wild-type; SKO, Seipin knockout*.

* *P < 0.05, compared with the WT*.

### AT Transplantation Could Not Rescue Cardiac Hypertrophy After TAC in SKO Mice

**Figure 6:**
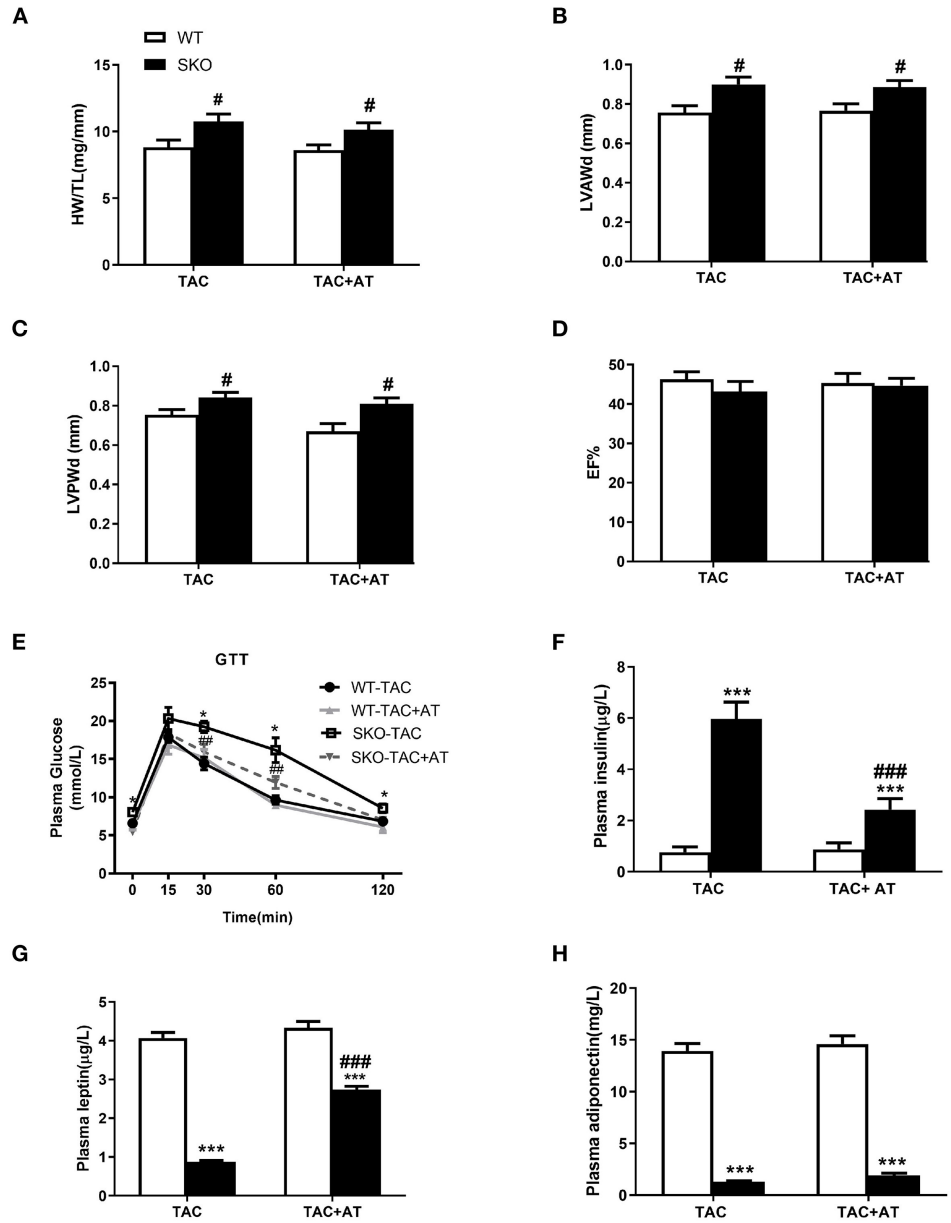
AT transplantation did not rescue the cardiac hypertrophy after TAC in *seipin* deficient mice and improved of insulin resistance. **(A)** Ratio of heart weight/tibia length (HW/TL); **(B)** Left ventricular anterior wall thickness in diastole (LVAWd); **(C)** Left ventricular posterior wall thickness in diastole (LVPWd); **(D)** Ejection fraction (EF %); **(E)** Glucose tolerance test (GTT) results; **(F)** plasma levels of insulin; **(G)** leptin; and **(H)** adiponectin. *P < 0.05, ***P < 0.001, effect of AT transplantation; ^#^*P* < 0.05, ^##^*P* < 0.01, ^###^*P* < 0.001, effect of genotype. AT, Adipose tissue; WT, Wild-type; SKO, *Seipin* knockout; TAC, transverse aortic constriction.

Previous studies have been shown that AT transplantation can effectively ameliorate hepatic steatosis and renal injury in SKO mice (16, 21). Therefore, in this study, we investigated whether transplantation of normal AT can ameliorate cardiac hypertrophy in SKO mice. More severe cardiac hypertrophy was observed in SKO mice than in WT mice at 12 weeks after TAC, the HW/TL and echocardiography results were similar to those we detected previously in this study. However, AT transplantation did not rescue cardiac hypertrophy in SKO mice at 12 weeks after TAC (Figures 6A–D).

GTT and plasma insulin levels indicated that SKO mice showed impaired glucose tolerance and hyperinsulinemia, and AT transplantation significantly improve these two parameters (Figures 6E,F). Loss of AT in SKO mice induced remarkably low plasma leptin levels and adiponectin, which are two important cytokines (Figures 6G,H; *P* < 0.001). Plasma leptin levels were rescued in SKO mice after AT transplantation (Figure 6G; *P* < 0.001), and plasma adiponectin levels remained unchanged after AT transplantation as described in the previous studies (Figure 6H).

## Discussion

In this study, we found that *Seipin* deficiency could promote cardiac hypertrophy and diastolic heart failure after TAC in mice. These changes may be related to the impairment of myocardial calcium handling, ER stress, inflammation, and apoptosis.

Previous clinical studies on *Seipin* deficiency have mainly focused on metabolic disorders. However, an increasing number of clinical studies have shown that *Seipin* mutation or deletion could lead to cardiac hypertrophy and heart failure, which is one of the leading causes of death in patients (3). However, the exact mechanism underlying the effect of *Seipin* on cardiac remodeling is not clear.

It is well-known that an imbalance in calcium homeostasis leads to heart failure (13, 28, 29). Therefore, we examined myocardial Ca^2+^ channel-related gene expression in this study. Compared with the sham-operated group, the SKO-TAC group showed decreased *Serca2a*. A previous study showed that SEIPIN has a close relationship with SERCA2a in adipocytes in Drosophila (14). A binding site of SERCA2a was found on the circular domain of human SEIPIN. The knockdown of *Seipin* in adipocytes in Drosophila inhibits the activity of SERCA2a, leading to an impaired Ca^2+^ transport from the cytosol to the endoplasmic reticulum and activation of endoplasmic reticulum stress metabolism, finally promotes lipid storage in Drosophila fat cells (14, 30).

Reduction of *Serca2a* plays an important role in diastolic heart failure in patients (31, 32). In this study, E/A and Em/Am were decreased significantly, and ventricular cavity enlargement indicated that the mice had diastolic dysfunction. These observations may be related to a decrease in the expression level of the SERCA2a (Figures 5A,D). In our *in vitro* experiments, it was found that the attenuation of Ca^2+^ transient currents in myocardial cells in SKO mice was slowed (Figure 5H), and the calcium influx decreased. A decrease in the amplitude of depleted cardiac calcium transient currents and reduced decay rates may be associated with slowing of the SERCA2a-mediated calcium transport. When SERCA2a function is inhibited, the transfer of Ca^2+^ to the endoplasmic reticulum is reduced, which may lead to a decrease in RYR (33, 34). In our study, the reduction of the *Ryr* gene expression in the hearts of SKO-TAC mice may be related to the decline in *Serca2a*.

SEIPIN is an ER membrane protein. As described previously, *Seipin* inhibits SERCA2a activity, leads to an impaired Ca^2+^ transport from cytosol to ER and actives ER stress metabolism in Drosophila fat cells (14). Another recent study has shown that the depletion of *Seipin* in the liver induces an increase in intracellular triglyceride *via* the activation of ER stress by influencing the intracellular calcium level (35). These two studies indicated that a deficiency of *Seipin* could induce ER stress due to impaired Ca^2+^ transport. In our study, we found that expanded sarcoplasmic reticulum and increased levels of ER stress related factors in the heart of SKO mice compared to WT mice after TAC (Figures 4A,D,G). ER stress plays an important role in heart failure. We suggested that increased ER stress induced by impaired Ca^2+^ transport aggravated heart failure in SKO mice. ER stress could also induce impaired Ca^2+^ transport. The causal relationship between the two factors needs further study.

In this study, myocardial apoptosis increased in SKO mice after TAC (Figures 4B,C). It has been reported that ER stress in the myocardium can cause apoptosis and inflammation of cardiomyocytes (36). Our study suggested that increased ER stress led to myocardial apoptosis in SKO mice.

Diastolic heart failure is usually related to metabolic cardiomyopathy (37). The SKO mice created by our group presented a severe loss of AT, fatty liver, and IR without hypertriglyceridemia (38). To study whether the adipokines play a role in diastolic heart failure due to *Seipin* deficiency, we transplanted AT from WT mice into SKO mice (16, 21). IR was rescued by AT transplantation as previously described (21). However, cardiac hypertrophy in SKO AT-transplanted mice was not rescued compared with that in untreated SKO mice after 3 months of TAC (Figure 6). Our results suggested that heart failure in SKO mice might be associated with mechanisms other than IR. Certainly we cannot rule out that the amount of transplanted fat was not sufficient to reverse heart failure, although the IR has been significantly improved with increasing plasma leptin levels. Hypoadiponectinemia in SKO mice was not improved by AT transplantation, and this result was confirmed by two previous studies (16, 39). A previous study has shown that lack of adiponectin exacerbates myocardial hypertrophy and leads to diastolic dysfunction (40). Therefore, hypoadiponectinemia might be involved in myocardial hypertrophy in the SKO mice. To clarify this problem, we could conduct replacement therapy with adiponectin in the SKO mice. Certainly, there are many additional adipokines that might also be involved in myocardial hypertrophy. The exact mechanisms require further investigation.

In a previous study, spontaneous cardiac hypertrophy and diastolic heart failure were found in 14-week-old SKO mice (41). This demonstrated that glucotoxicity with increased myocardial glucose uptake can trigger cardiac dysfunction. In our experiments, SKO mice showed no cardiac hypertrophy at 4 months, as was demonstrated in a previous study (38), and showed only mild cardiac hypertrophy at 9–10 months. These results are different from those of the Joubert et al. study (41). A review summarized the diverse phenotypes of SKO mice in the different groups in terms of body weight, food intake, plasma glucose, and organomegaly. The SKO mice created by our group had mild hyperglycemia (160 mg/dl) in fasted conditions and showed no difference after feeding (42). The exact mechanisms are unknown. No glycogen or lipid deposition in the myocardium was found in SKO-TAC mice (results not shown). In autopsies of patients with *Seipin* gene mutations or defects, the diseased myocardial tissue showed no deposition of glycogen and lipids (43, 44).

SKO mice manifest severe hepatic steatosis (21, 38), whereas liver-specific SKO mice do not exhibit hepatic steatosis (45). Interestingly, AAV-mediated *Seipin* overexpression in the liver alleviated high-fat diet-induced hepatosteatosis in mice by increasing mitochondrial activity (35). A study has shown that no heart failure was found in cardiac-specific *Seipin* gene deficient mice (46). It would be interesting to know whether *Seipin* overexpression in the heart can improve cardiac hypertrophy, and this might provide particularly valuable evidence on *Seipin* gene function in cardiac hypertrophy.

The PI3K/AKT and JAK/STAT3 signaling pathways were activated in the heart tissue of SKO mice (46, 47). Chronic activation of the p38 MAPK pathway associated with apoptotic cell death was observed in the *Seipin*-knockdown adipocytes (48). The JNK pathway was activated in the hippocampus of the SKO mice (49). It would be interesting to know whether the p38 MAPK and CHOP/JNK pathways are involved in cardiac hypertrophy in SKO mice. The exact mechanisms should be further investigated.

In conclusion, we demonstrated that *Seipin* gene deficiency induced decreased SERCA2a and impaired Ca^2+^ transport, increased ER stress, myocardial inflammation, and apoptosis in heart, and finally increased diastolic heart failure. This experiment provides clues for studying the mechanism of heart failure caused by *Seipin* gene defects. The initial cause needs further study.

## Data Availability Statement

The original contributions presented in the study are included in the article/supplementary material, further inquiries can be directed to the corresponding author.

## Ethics Statement

The animal study was reviewed and approved by Institutional Animal Care Research Advisory Committee of Peking University Health Science Center.

## Author Contributions

XW and WH designed the project. WH supervised the project. XW, XL, HW, ZZ, LZ, and XS performed the experiments. XW and XL analyzed the data and wrote the manuscript. CY, ZL, YW, and YZ provided technical advice. XW, XX, GL, and WH revised the manuscript. All authors contributed to the article and approved the submitted version.

## Conflict of Interest

The authors declare that the research was conducted in the absence of any commercial or financial relationships that could be construed as a potential conflict of interest.
